# *Alhagi pseudalhagi* Extract Exerts Protective Effects Against Intestinal Inflammation in Ulcerative Colitis by Affecting TLR_4_-Dependent NF-κB Signaling Pathways

**DOI:** 10.3389/fphar.2021.764602

**Published:** 2021-11-04

**Authors:** Xiaoqin Xu, Juan Zhang, Liang Chen, Yu Sun, Degang Qing, Xuelei Xin, Chunyan Yan

**Affiliations:** ^1^ State Key Laboratory Basis of Xinjiang Indigenous Medicinal Plants Resource Utilization, Key Laboratory of Plant Resources and Chemistry in Arid Regions, Xinjiang Technical Institute of Physics and Chemistry, Chinese Academy of Sciences, Urumqi, China; ^2^ Xinjiang Institute of Chinese Materia Medica and Ethnodrug, Urumqi, China; ^3^ University of Chinese Academy of Sciences, Beijing, China; ^4^ Affiliated Traditional Chinese Medicine Hospital of Xinjiang Medical University, Urumqi, China; ^5^ Guangdong Pharmaceutical University, Guangzhou, China

**Keywords:** *Alhagi pseudalhag*i, ulcerative colitis, intestinal inflammation, anti-inflammatory, TLR4-dependent NF-κB signaling pathways

## Abstract

*Alhagi pseudalhagi* Desv. Extract (APE) is the major active fraction extracted from the aerial part of *Alhagi pseudalhagi* Desv. In view of its application in Uyghur medicine, it may be beneficial for the treatment of ulcerative colitis (UC). The aim of the present study was to investigate the possible beneficial effects of APE on UC mice and detect the possible mechanisms underlying these effects.

**Methods:** An acute UC model was established in mice using dextran sulfate sodium. Sixty mice were randomly divided into six groups: normal, UC model, sulfasalazine (200 mg/kg), high-dose APE (APE-H, 2.82 g/kg), middle-dose APE (APE-M, 1.41 g/kg), and low-dose APE (APE-L, 0.70 g/kg) groups. Drugs were administered by gavage for 10 days after the induction of colitis. Serum and colon tissue samples were collected from the mice during the experiment, and survival signs, body weight changes, disease activity index (DAI), colon length, and colon wet weight in mice were determined after the treatment. UC-induced damage, including inflammation and ulceration of colon mucosa, were observed by the naked eye as well as using hematoxylin and eosin staining (H&E) and scanning electron microscopy and scored according to Wallace and Keean’s criteria. We measured the levels of tumor necrosis factor α (TNF-α), interleukin (IL)-1β, IL-6, and IL-10 in the serum and colon tissues using ELISA. Additionally, the relative protein levels of toll-like receptor 4 (TLR_4_), nuclear factor-kappa B p65 (NF-κB p65), phosphorylated NF-κB p65 at Ser536 (p-p65 Ser536), inhibitor kappa B-kinase ß (IK-Kβ), and phosphorylated IK-Kβ (Ser176/180) (p-IK-Kβ) in colonic mucosal epithelial tissues were detected using western blotting. The main functional components of APE were analyzed and confirmed by UPLC-MS/MS.

**Results:** APE treatment repaired the UC-induced colon mucosa injury, reduced the weight loss, attenuated DAI, colon macroscopic damage index, and histological inflammation, and significantly downregulated the levels of inflammatory markers, including TNF-α, IL-1β, and IL-6, in the serum and colon tissues. Additionally**,** APE treatment reduced the levels of TLR_4_ and phosphorylation of p-NF-κB and p-IK-Kβ. The main components of APE are taxifolin, 3,5-dihydroxy-2-(4-hydroxyphenyl)-7-[(2R,3R,4S,5S,6R)-3,4,5-trihydroxy-6-(hydroxymethyl) oxan-2-yl] oxychromen-4-one, hyperoside, rutin, kaempferol, isorhamnetin, 7,8-dihydroxyflavone, and kaempferide.

**Conclusions:** To the best of our knowledge, the present study is first to demonstrate that APE exerts a protective effect against intestinal inflammation in UC by affecting TLR_4_-dependent NF-κB signaling pathways.

## Introduction

Ulcerative colitis (UC) is a chronic and recurrent gastrointestinal disease (Xavier and Podolsky, 2007), with an increasing yearly incidence (Frolkis et al., 2013). UC is characterized by reccuring difficulty in healing (Molodecky et al., 2012) and carcinogenesis (Jiang and Cui, 2008). Its clinical manifestations include diarrhea, abdominal pain, and mucinous blood stools (Yang et al., 2018). Patients with UC are prone to toxic colonic dilation, intestinal perforation, hemorrhage, hypothrombin, polyps, cancer, and complications related to autoimmunity, which seriously threaten public health (Pitchumoni and Chari, 2013). UC may be related to inflammation, immune disorders, infection, genetic susceptibility, epithelial barrier defects, adverse environmental factors, lifestyle, and diet habits (Ungaro et al., 2017). Multiple pathogeneses have been demonstrated in the occurrence and development of UC, among which inflammatory factors play a crucial role (Cosnes et al., 2011; Roselli and Finamore, 2020). Recent studies have suggested that mucosal immune deficiency cannot effectively reduce inflammation, which may be the key factor leading to intestinal inflammatory disease. Various cells, cytokines, and inflammatory media form a complex network system, leading to chronic inflammation in UC through multiple signaling pathways. The toll-like receptors/nuclear factor-kappa B (TLR_S_/NF-κB) pathway is one of the hotspots in current research. Studies have shown that TLR_4_ is slightly expressed in normal intestinal epithelial cells but highly expressed in the mucosa of ulcerative colitis (Reaves et al., 2005), indicating that the TLR_4_-mediated signal transduction pathway is an important link in the pathogenesis of UC (Zhang et al., 2014). NF-κB is an important signal transduction, cell activation, and transcriptional activator in the downstream signaling pathway of the TLR_4_ immune regulatory network (Li et al., 2011), which regulates the levels of cytokines IL-1, IL-6, TNF-α, adhesion molecules, immune receptors, pro-apoptotic, and anti-apoptotic proteins; therefore, NF-κB induces inflammatory immune responses (Wu et al., 2016).

In the past 2 decades, much progress has been made in UC diagnosis and therapy. Therapeutic drugs, including aminosalicylic acids, glucocorticoids, and immunosuppressants, can relieve UC symptoms; however, they display some side effects. Therefore, an effective therapeutic drug is yet unavailable. Traditional Chinese medicine for the treatment of UC has the advantages of clinical dialectics and comprehensive treatment, and the discovery of natural products or compound drugs that regulate intestinal inflammation from traditional Chinese medicine has gradually become the center of research into UC drugs.

*Alhagi pseudalhagi* Desv is a component of Uyghur traditional medicine, distributed in arid and semi-arid areas, and named due to it being a preferred food among camels. It is drought resistant, heat resistant, and sand resistant (Liu, 1985). It has a unique chemical composition and pharmacological activity with respect to its special habitat. It has been used for clearing away heat and detoxification, reducing swelling, and relieving pain. It also relieves abdominal pain and distension, dysentery, and diarrhea by nourishing, strengthening, and balancing bodily fluids (Li, 1996; Liu, 1999). A previous study found that *Alhagi pseudalhagi* extracts (Xu et al., 2012; Xu et al., 2016) regulate the intestinal movement of experimental mice and guinea pigs *in vitro* and *in vivo* (Jia et al., 2009; Abuliz et al., 2013; Mikeremu et al., 2017), and could alleviate abdominal pain, diarrhea, and bloody stools, symptoms consistent with that of UC. Our previous study confirmed that the *A. pseudalhagi* extract had a certain inhibitory effect on the release of tumor necrosis factor-α (TNF-α) (Zhang and Zhang, 2010). TLR_4_-dependent NF-κB is the regulatory signaling pathway of TNF-α, IL-6, and other genes, and is closely associated with inflammatory responses (Hasnat et al., 2015). However, it remains unknown whether the *A. pseudalhagi* Desv extract exerts its anti-inflammatory and protective effect in UC therapy. Therefore, in this study, we aimed to explore the protective effect of APE on colonic mucosal injury and its possible mechanism, so as to provide a new strategy for the prevention and treatment of UC.

## Materials and Methods

### Animals

Male ICR mice of SPF grade (6–8 weeks old, 18–22 g) were purchased from the Animal Center of Xinjiang Medical University [SYXK (Xin) 2018-0003]. During the experiment, the mice were housed in a 12 h dark/light circulating environment at room temperature (22 ± 3°C), relative humidity of 60–80%, and free access to standard diet and purified water. Animal welfare and experimental procedures were carried out in strict accordance with the “Guidelines for the Management and Use of Laboratory Animals” (Ministry of Science and Technology of China, 2006) and approved by the Animal Ethics Committee of Xinjiang Medical University and the Animal Protection and Utilization Committee.

### Chemicals

The herb Alhagi was obtained from the Turpan Depression (Xinjiang, China) and identified by Professor Yonghe Li (College of Traditional Chinese Medicine, Xinjiang Medical University, Xinjiang, China). Specimens of the herbs (NO: LTC_20190612) were stored in the herbarium center of the Xinjiang Institute of Chinese MateriaMedica and Ethnodrug. Dextran sulfate sodium (DSS; No.0216011080, 36,000–50,000Da) was purchased from MP Biomedicals and dissolved in 0.9% NaCl to a final 2.5%. Chromatographic grade methanol, acetonitrile, and formic acid were obtained from CNW Technologies (Shanghai, China). Sulfasalazine (SASP) was purchased from Sigma Aldrich (St. Louis, MO, United States). SuperSignal™ West Pico PLUS and Chemiluminescent Substrate were purchased from Thermo Fisher Scientific. Antibodies against TLR_4_ (Cat.No.bs-20594R), NF-κBp65 (Cat.No.bs-0465R), p-NF-κB p65 (Ser536) (Cat.No.bs-0982R), and IK-Kβ (Cat.No.bs-4880R) were purchased from Beijing Boaosen Biotechnology Co., Ltd. (Beijing, China). The antibody *p*-IKKα/β (Ser176/180) (Cat. No. 2694S) was purchased from Cell Signaling Technology. Goat Anti-Mouse or Anti-Rabbit IgG was purchased from Abcam. A mouse IL-1β, IL-6, IL-10, and TNF-α ELISA kits were purchased from Lianke Biotechnology Co., Ltd. (Xinjiang, China).

### Ultra-Performance Liquid Chromatography–Tandem Mass Spectrometry Analysis of Extract

We added 100 mg of sample to 1,000 μl of extracted solution containing 10 μg/ml of internal standard dissolved in 80% methanol. The samples were homogenized at 45 Hz for 4 min and sonicated for 1 h in an ice-water bath (−20°C). Subsequently, the samples were centrifuged at 12,000 rpm for 15 min at 4°C. Finally, the supernatant was obtained and put in a fresh 2 ml tube for UPLC-MS/MS analysis.

UPLC-MS/MS analysis was performed on an ultra-high performance liquid chromatography vanquish system (Thermo Fisher Scientific) with a Waters UPLC BEH C_18_ column (1.7 μm, 2.1 × 100 mm). The UPLC conditions were as follows: flow rate, 0.4 ml/min; sample injection volume, 5 μl; and total running time, 30 min. The mobile phase consisted of 0.1% formic acid in water (A) and 0.1% formic acid in acetonitrile (B). The multi-step linear elution gradient program was as follows: 0–3.5 min, 95–85% A; 3.5–6 min, 85–70% A; 6–6.5 min, 70–70% A; 6.5–12 min, 70–30% A; 12–12.5 min, 30–30% A; 12.5–18 min, 30–0% A; 18–25 min, 0–0% A; 25–26 min, 0–95% A; and 26–30 min, 95–95% A.

An Q Exactive Focus mass spectrometer coupled with an Xcalibur software was employed to obtain the MS and MS/MS data based on the IDA acquisition mode. During each acquisition cycle, the mass range was from 100 to 1,500, and the top three of every cycle were screened and the corresponding MS/MS data were further acquired. The MS conditions were as follows: sheath gas flow rate: 45 Arb, aux gas flow rate: 15 Arb, capillary temperature: 400°C, full MS resolution: 70,000, MS/MS resolution: 17,500, collision energy: 15/30/45 in NCE mode, and spray voltage: 4.0 kV (positive) or 3.6 kV (negative).

### UC Model Preparation and Treatment Procedure

**FIGURE 1:**
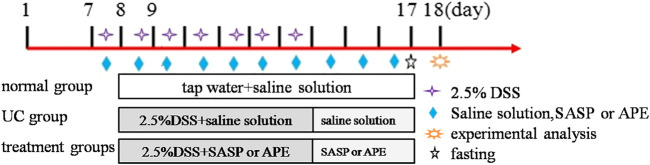
The experimental design. 1–7 days are adaptive feeding stage, 8–17 days are experimental stage.

After 1 week of adaptive feeding, unhealthy animals were excluded. According to a random number table, the healthy animals (*n* = 60) were randomly divided into the following six groups, with ten mice in each group: normal control (NG, saline solution daily), UC model (UG, 2.5% DSS solution), sulfasalazine (SASP) control (SG, 0.2 g/kg), and high-dose APE (APE-H, 2.82 g/kg), middle-dose APE (APE-M, 1.41 g/kg), and low-dose APE (APE-L, 0.70 g/kg) groups. During the experiment periods, except for the NC group, the mice of other experimental groups were administered 2.5% DSS (w/v) in drinking water for seven consecutive days to induce an acute UC model in mice (Chin et al., 2018). The DSS solution was replaced every day. At the same time of modeling, each treatment group was given the corresponding therapeutic drugs orally, once a day for 10 consecutive days. The volume of intragastric administration was 0.1 ml/10 g body weight. Mice were given a normal diet during the whole experiment. In the study, we used the equivalent dose calculated based on the acute toxicity test results of mice from the experiment doses. The experimental low doses were 1/20 of the maximum doses. The maximum dose of APE was 8.08 g/kg and the APE extraction ratio was 8.69%; therefore, the low dosage in mice was 8.08 g × 8.69 % ≈ 0.70 g/kg. Low dose increased by two times as APE-M and increased by four times as APE-H. On the ninth day of the experiment, all groups of mice fasted without water, their peripheral blood (orbital blood) was collected, and all mice were intraperitoneally injected with 1% amobarbital sodium. Furthermore, 24 h after the last administration, the colon was excised for the following experiments. The experimental design is shown in Figure 1.

The successful construction of the UC model in mice was evaluated based on the following criteria: on the second day of model preparation, mice experienced diarrhea of different degrees and increased stool frequency, and the stool was septic and bloody.

### Measurement of Disease Activity Index, Colon Length, and Wet Weights

The clinical assessment of disease severity was evaluated using DAI scores which mainly comprised weight loss percentage, stool consistency, and fecal bleeding. The colonic length and wet weight of each group were measured and analyzed. DAI = (score of weight loss + stool consistency + fecal bleeding)/3 (Cooper et al., 1993). The details are shown in Table 1.

**TABLE 1:** Disease activity index(DAI) score.

Score	Weight loss(%)	Stool consistency	Fecal bleeding
0	None	Well-formed pellets	Normal
1	1–5	Loose stools	Constipation
2	6–10		
3	11–15		
4	>15	Diarrhea	Bloody stools

### Observation of Colon Macroscopic Damage Index

The animals fasted for 24 h and were anesthetized by intraperitoneal injection of 1% sodium isoprenobarbital (0.4 ml/100 g) and the colon tissue (from ileocecal junctions to anal edge) were immediately resected. Further, the mesenteric tissue, blood vessels, fat, and feces were removed, and measured by blind evaluation via CMDI involving severity of inflammation and existence of ulcer scored according to Wallace and Keean’s criteria (Wallace and Keenan, 1990). The scoring criteria were described as follows: 0, no mucosa injury, no adhesion; 1, slight hyperemia edema, no ulcer, no erosion; 2, presence of adhesions and a slight ulcer; 3, presence of ulcers and inflammation; 4, marked ulcers and increased inflammation; and 5, significant increase in the area of ulcer and inflammation.

### Histological Analysis of the Colon

Five mice colon samples from each group were selected by random choice, and then the samples of colonic tissue were fixed in 4% paraformaldehyde and embedded in paraffin. The embedded colon tissues were cut into 5 μm slices and stained with hematoxylin and eosin (H and E). The sections were detected under a microscope (E200, Nikon, Japan) by a researcher blinded to the treatments. The number of infiltrating inflammatory cells, ulcers, and bleeding and necrotic cells were calculated. The severity of colon tissue damage was assessed by using the pathological score (Cooper et al., 1993) (Table 2).

**TABLE 2:** Histopathological score of colon.

Score	Inflammation	Crypt destruction	Lesion depth	Lesion range %
1	Slightly	basal 1/3 is destroyed	mucosal layer	1–25
2	Moderate	basal 2/3 was destroyed	submucosa	26–50
3	—	cases with only intact surface	epithelium	51–75
4	Severe	All crypt and epithelium were destroyed	Serosal layer	76–100

### Measurement of Biochemical Indices in Colon Tissues and Serum

Peripheral blood (1–2 ml) collected using the orbital blood collection method and centrifuged at 4,000 rpm and 4°C for 10 min. The plasma supernatant was collected, the ground tissue sample was homogenized with normal saline, and 100 μl of the original solution was used for ELISA measurement. The levels of tumor necrosis factor α (TNF-α) (EK282/3-96), interleukin (IL)-1β (EK201B/3-96), IL-6 (EK206/3-96), and IL-10 (EK210/3-96) were measured by using the ELISA kits from Lianke Biotechnology Co., Ltd. (Xinjiang, China). All biochemical indices were measured on a microplate (XMarkTM, Bio-Rad, United States)

### Western Blot Analysis

Anti-p-NF-κB P65 (1:800, bs-0982R), anti-TLR_4_ (1:1,000, bs-20594R), anti-IKK, TLR_4_, NF-κB p65, NF-κB p-p65 (Ser536), IK-kβ, and p-IK-kβ (Ser176/180) protein expressions in colon tissues were detected using western blotting. Colon tissues were ground in liquid nitrogen; approximately 100 mg of the tissue sample was added to a precooled 1.5 ml centrifuge tube along with 400 μl RIPA lysis buffer (protease inhibitor and broad-range phosphatase inhibitor) and thoroughly mixed. After the samples were placed at 4°C for 60 min, the supernatant that has been centrifuged at 12,000 rpm at 4°C for 15 min, was collected. Protein concentrations were quantified using the BCA kit. Relative protein levels were calculated by using internal reference ß-actin. Equal amounts of protein (50 μg) of each sample were separated by sodium dodecyl sulfate-polyacrylamide gel electrophoresis using 10% polyacrylamide gels and electrophoretically transferred to polyvinylidene fluoride membranes in the transfer buffer at 100 V for 90 min (TLR_4_, IK-Kβ, and P-IK-Kβ) or 60 min (P65, P-P65, and actin). Blocking solution containing 5% skim milk powder was used to seal the transfer film for 1 h, and the membrane was washed with TBST thrice for 5 min each. The membranes were then incubated with the following primary antibodies: anti-β-actin (1:1,000, Santa Cruz Biotechnology), anti-NF-κB P65 (1:800, bs-0465R, Bioss Biotechnology Co., Ltd., ß (1:1,000, bs-4880R) and IKKβ (Ser176/180) (1:1,000, 2694S) overnight at 4°C. Blots were rinsed three times with TBST (Solarbio, China), and then incubated with horseradish peroxidase (HRP)-conjugated secondary antibodies for 2 h at 37°C. Relative protein levels were calculated by Chemiscope 3,000 (Shanghai Qinxiang Scientific Instrument Co., Ltd., China).

### Statistical Analyses

Data are presented as the means ± standard deviation. Statistical analyses were performed with SPSS19.0 software. One-way analysis of variance (ANOVA) with post-hoc Tukey’s tests were used to evaluate the variables between groups. The LSD method was used for the pairwise comparison between groups. A non-parametric test of multiple independent samples was used for the comparison of pathological scores. Differences with *p* < 0.05 or *p* < 0.01 were considered statistically significant.

## Results

### Identification of the Major Components of APE

**FIGURE 2:**
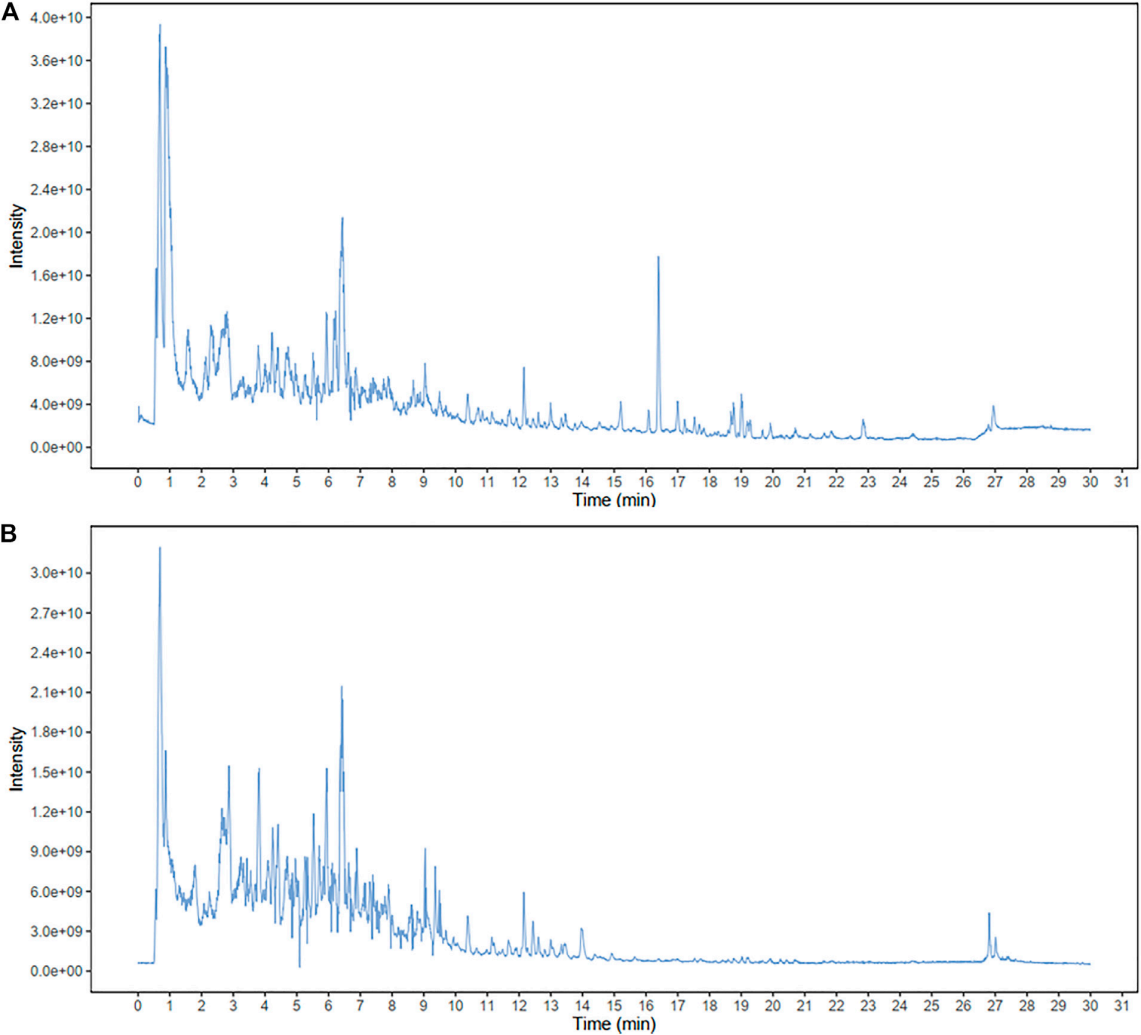
Identification of major components of APE examined by UPLC-MS/MS.The positive **(A)** and negative **(B)** ion chromatograms of APE are shown as indicated. APE, *Alhagi pseudalhagi* extract.

The total flavonoid content of APE, analyzed using the ultraviolet spectrophotometer method, was 16.94%. To identity the main ingredients, APE samples were evaluated by UPLC-MS/MS. The total positive (Figure 2A) and negative (Figure 2B) ion chromatograms of APE demonstrated the composition of all ingredients. We obtained the retention time of the ion flow pattern and the information of the mass spectrum parameters and analyzed the rule of mass spectrum breakdown in each ion flow peak. Compounds were identified by referring to the Biotree TCM mass spectrometry database. UPLC-MS/MS analysis demonstrated the elution time of the main APE compounds as follows: taxifolin, 3.643 min; 3,5-dihydroxy-2-(4-hydroxyphenyl)-7-[(2R,3R,4S,5S, 6R)-3,4,5-trihydroxy-6-(hydroxymethyl) oxan-2-yl] oxychromen-4-one, 5.409 min; hyperoside, 5.914 min; rutin, 5.924 min; kaempferol, 6.267 min; isorhamnetin, 7.252 min; 7,8-dihydroxyflavone, 7.529 min; and kaempferide, 9.915 min.

### APE Ameliorate DSS-Induced Colitis

**FIGURE 3:**
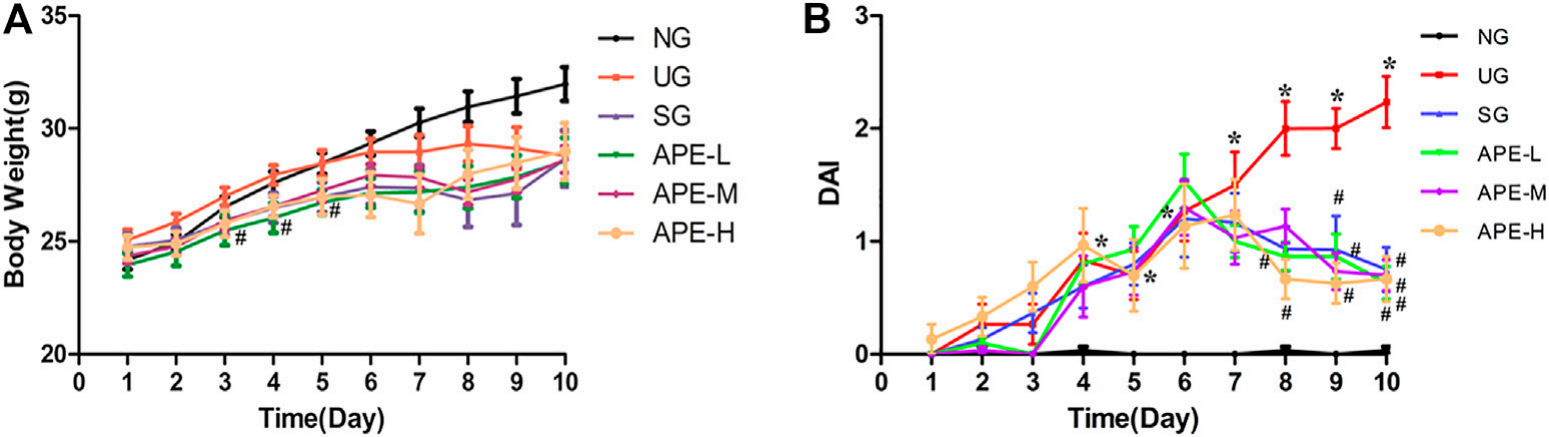
Effects of APE on the body weight **(A)** and DAI **(B)** in each group. *n* = 10 for each group.*p < 0.05, **p < 0.01 vs. NG.^#^ *p* < 0.05, ^##^ *p* < 0.01 vs. UG.APE, *Alhagi pseudalhagi* extract; DAI, disease activity index; NG, normal group; UG, ulcerative colitis model group; APE-H, high-dose APE group; APE-M,middle-dose APE group; APE-L, low-dose APE group.

We observed the clinical features of UC in mice, including weight loss percentage, stool consistency, and fecal bleeding. From days 1–7 after DSS induction, the body weight of mice in each group showed a slow increase. The weight gain in the UG group was not as obvious as that in the NG group. Bloody stools and weight loss in the SG and APE groups were alleviated on day 6. The stools were better formed and the body weight of SASP and the APE administered groups rapidly increased from day 8. The decrease in body weight after DSS administration was gradually reversed by the APE treatment. There was no significant difference in body weight between the three APE groups (Figure 3A). Moreover, DAI in the UG group increased gradually compared with that in the NG group. After APE intervention, the DAI scores of mice decreased significantly (Figure 3B, *p* < 0.05), and the DAI scores of mice in the APE-H group decreased significantly compared with the APE-L and APE-M groups.

### APE Treatment Increased Colon Length and Decreased CMDI

**FIGURE 4:**
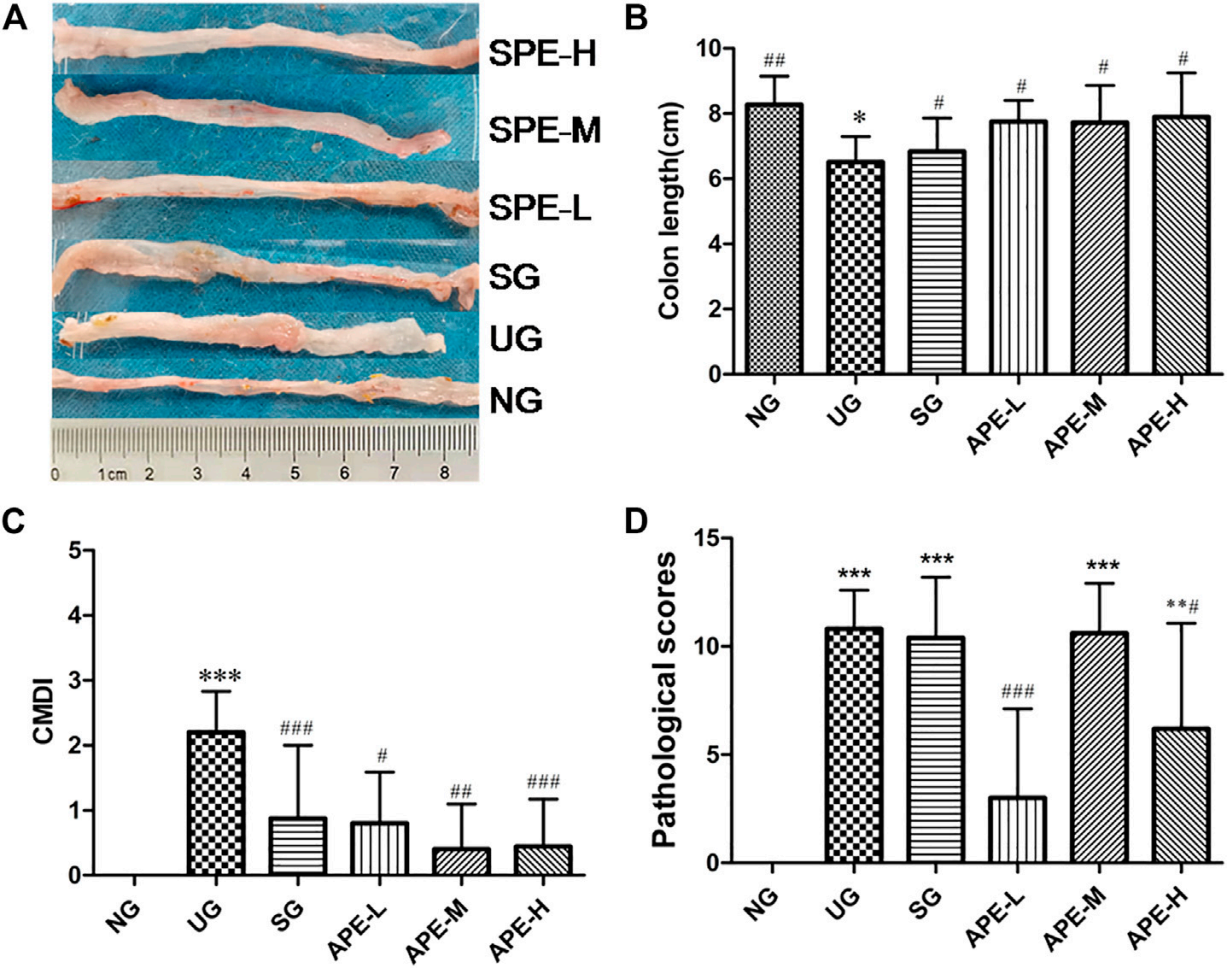
Effects of APE on colon length, CMDI and pathological scores. **(A)** Representative images of colon sections from mice in each group. **(B)** Quantification of the change in colon length in each group. **(C)** The change of CMDI in each group. **(D)** The pathological scores in each group. *n* = 10 for each group.**p < 0.01,***p < 0.001vs.NG. ^#^ *p* < 0.05, ^##^ *p* < 0.01, ^###^ *p* < 0.001 vs. UG.APE, *Alhagi pseudalhagi* extract; CMDI, colon macroscopic damage index; NG, normal group; UG, ulcerative colitis model group; APE-H, high-dose APE group; APE-M, middle-dose APE group; APE-L, low-dose APE group.

As shown in Figure 4A, compared with the NG group, the colon length in the UG group decreased significantly (Figure 4B, *p* < 0.05). APE treatment prevented DSS-induced colon length reduction in the UC mice, with statistically significant differences between the UG and the APE-H, APE-M, APE-L treatment groups (Figure 4B, *p* < 0.05). Compared with the UG group, colonic wet weight of mice in the SASP, APE-L, and APE-H groups showed a decreasing trend; however, there was no marked difference in colonic wet weight between the UG group and all the APE groups.

After the establishment of the UC model, the colon of the UG group showed obvious edema, hyperemia, inflammation, and ulcer. The CMDI scores were increased in the UG group when compared with the NG group (Figure 4C, *p* < 0.05). Ulcers, inflammation, and adhesions developed in the UG group mice after they were administered the DSS solution. APE treatment significantly alleviated the above damaged signs, and thus reduced the CDMI (Figure 4C, APE-H vs. UG *p* < 0.001; APE-M vs. UG *p* < 0.01; APE-L vs. UG *p* < 0.05). The APE-L group had a statistically significant difference compared with the APE-M group (Figure 4C, *p* < 0.001).

### APE Treatment Reduced Pathological Scores of UC

The colonic pathological scores of mice were higher in the UG group than in the NG group (Figure 4D, *p* < 0.05). The APE treatment reduced the pathological scores. There was an obvious difference between the APE-L, APE-H, and APE-M groups (Figure 4D, APE-L vs. UG *p* < 0.001, APE-H vs. UG, *p* < 0.05).

**FIGURE 5:**
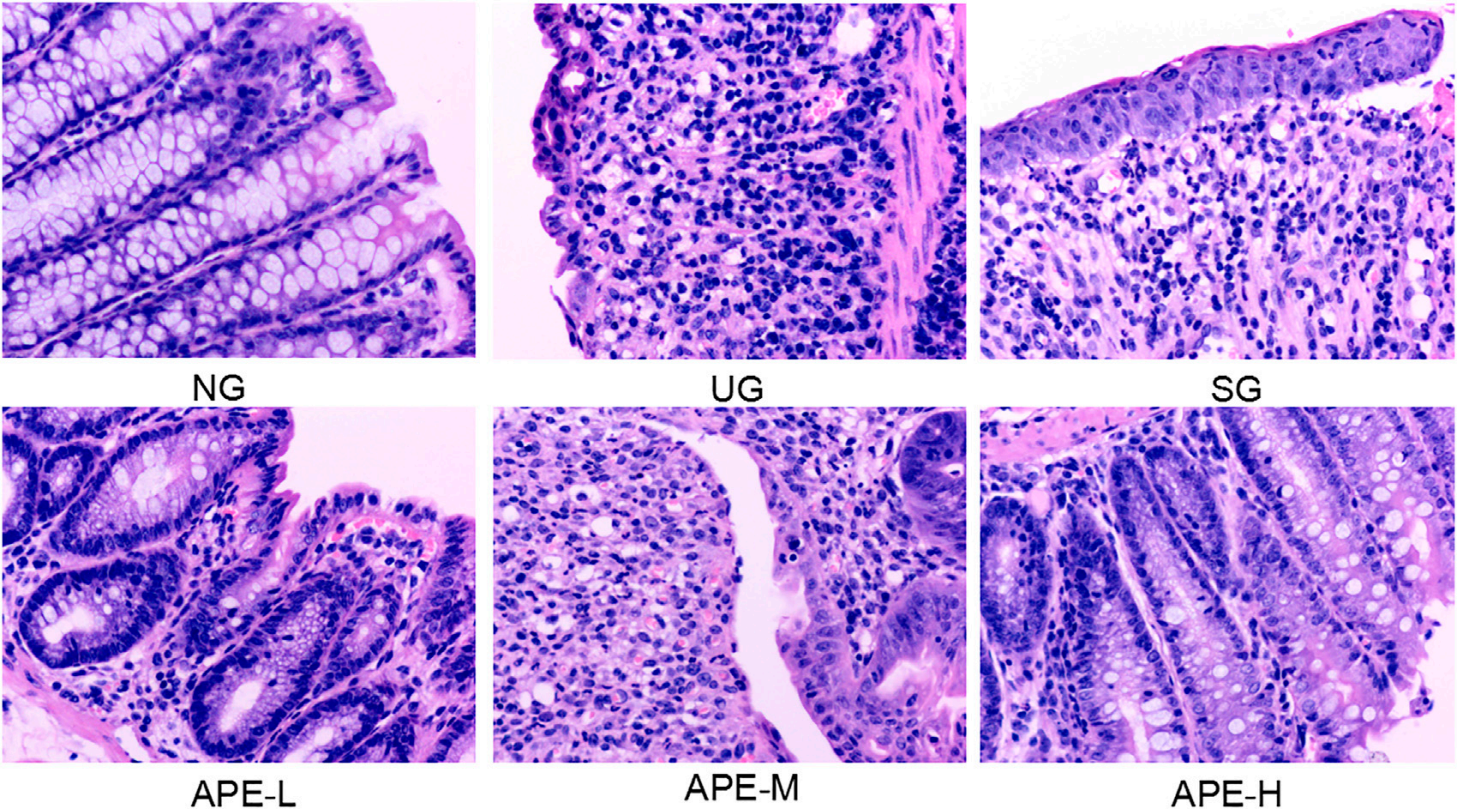
Effects of APE on the pathological alterations in the colon. Representative images of hematoxylin and eosin stained sections (H and E staining, 200×). APE, *Alhagi pseudalhagi* extract.

DSS induced pathological changes in the colon tissue, with large areas of erosion, more necrosis and exudation of tissue, hyperplasia of fibrous granulation tissue (ulcer formation), and partial glandular structure loss in the lamina propria. A large number of acute and chronic inflammatory cells (neutrophils, lymphocytes, plasma cells, and monocytes) infiltrated into the tissue, and crypt abscess were observed in some intestinal glands. Goblet cells were reduced in some of the glands. Low-grade intraepithelial neoplasia of focal epithelial cells was observed. After APE treatment, compared with the UG group, the area of colonic inflammation and ulcer in the APE-L and APE-H groups were reduced, the degree of colonic epithelial mucosal ulcer and the infiltration of lamina propria inflammatory cells were significantly reduced, the cryptum abscess in the intestinal gland gradually disappeared, the structure of the lamina propria gland was found to be complete (Figure 5), and the histological score was decreased (Figure 4D).

### APE Treatment Regulated the Expression of Inflammatory Cytokines in UC Mice

**FIGURE 6:**
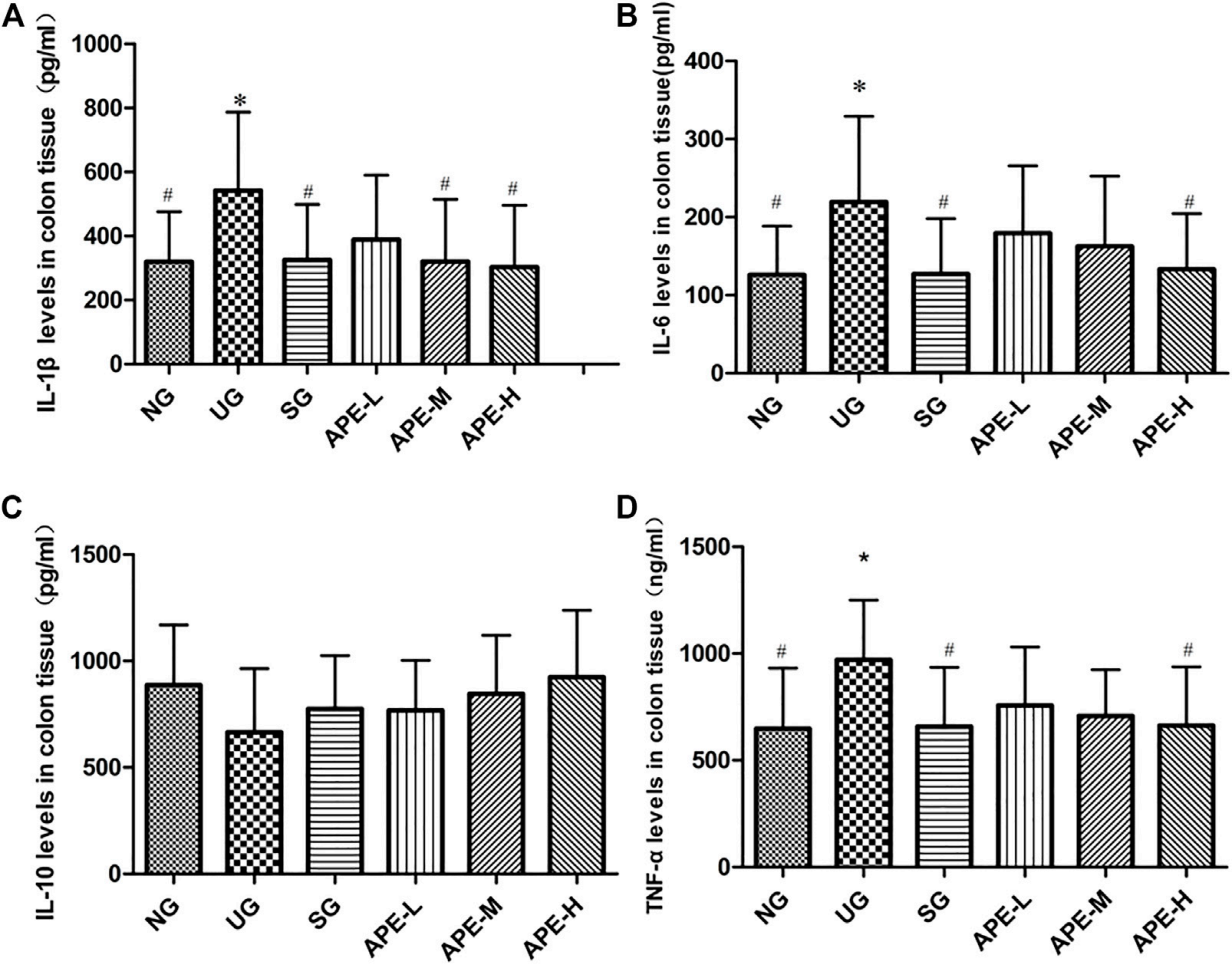
Effects of APE on the colon tissue levels of inflammatory factors in the UC mice. **(A)** IL-1β. **(B)** IL-6. **(C)** IL-10. **(D)** TNF-α. *n* = 8 for each group.*p < 0.05,**p < 0.01 vs. NG.^#^ *p* < 0.05, ^# #^ *p* < 0.01 vs. UG.APE, *Alhagi pseudalhagi* extract; UC, ulcerative colitis; IL-1β, interleukin-1β; IL-6, interleukin-6; IL-10, interleukin-10; TNF-α, tumor necrosis factor-α; NG, normal group; UG, ulcerative colitis model group; APE-H, high-dose APE group; APE-M, middle-dose APE group; APE-L, low-dose APE group.

**FIGURE 7:**
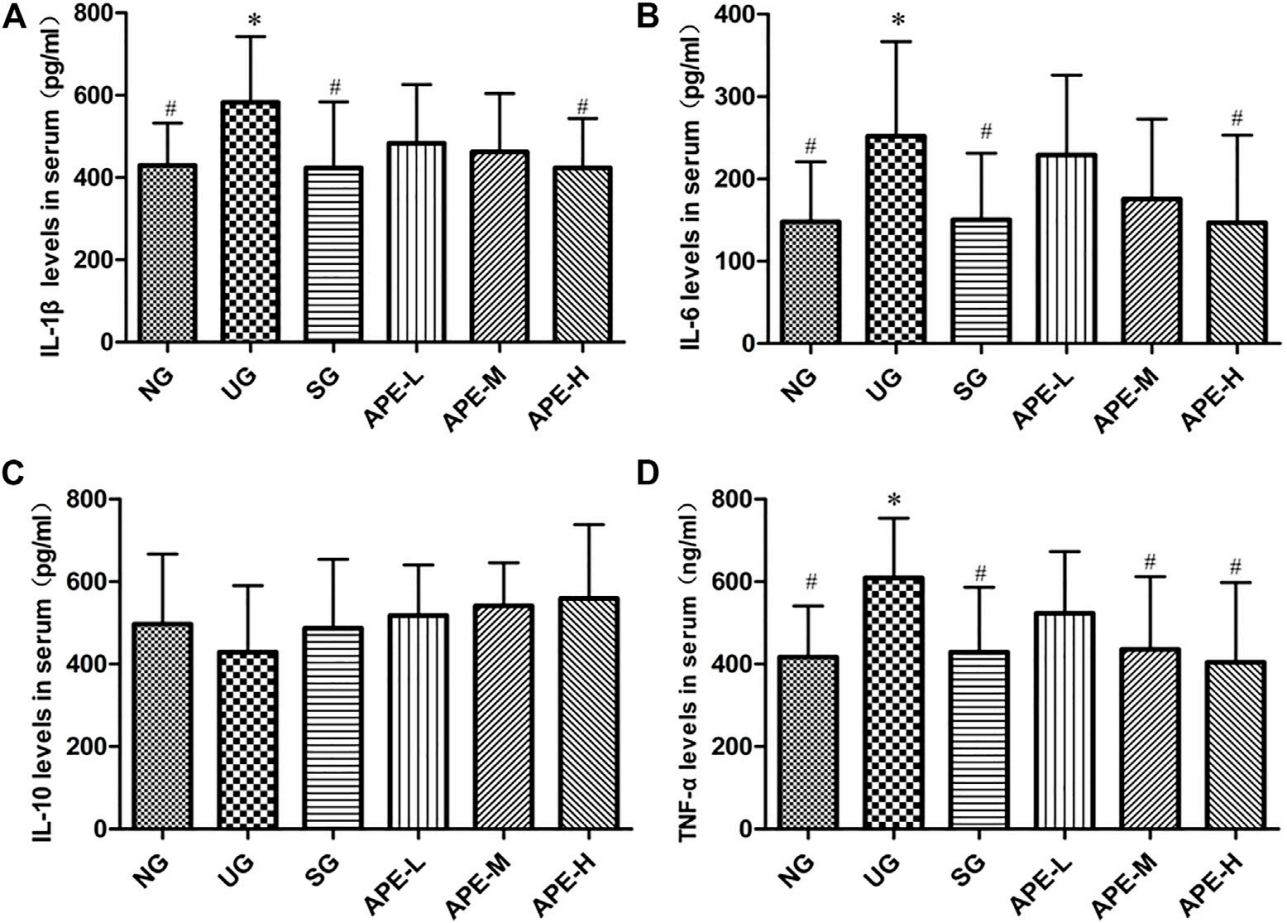
Effects of APE on the serum levels of inflammatory factors in the UC mice. **(A)** Interleukin-1β (IL-1β). **(B)** Interleukin-6 (IL-6). **(C)** Interleukin-10 (IL-10). **(D)** Tumor necrosis factor-α (TNF-α). *n* = 8 for each group.*p < 0.05, **p < 0.01 vs. the Normal group (NG).^#^ *p* < 0.05, ^# #^ *p* < 0.01 vs. the UC model group (UG). APE, *Alhagi pseudalhagi* extract; UC, ulcerative colitis; IL-1β, interleukin-1β; IL-6, interleukin-6; IL-10, interleukin-10; TNF-α, tumor necrosis factor-α; NG, normal group; UG, ulcerative colitis model group; APE-H, high-dose APE group; APE-M, middle-dose APE group; APE-L, low-dose APE group.

IL-1β, IL-6, and TNF-α levels were significantly higher in the colon tissue and serum of the UG group than in the NG group (Figure 6, IL-1β, IL-6, and TNF-α in colon tissue, UG vs. NG, *p* < 0.05; Figure 6, IL-1β, IL-6, and TNF-α in serum, UG vs. NG, *p* < 0.05). The IL-10 level decreased after induction by DSS in the UG group. More importantly, APE treatment significantly reduced IL-1β, IL-6, and TNF-α levels in colon and serum of UC mice (Figure 6, IL-1β, IL-6, and TNF-α in colon, APE-H vs. UG, *p* < 0.05; Figure 7, IL-1β, IL-6, and TNF-α in serum, *p* < 0.05) and enhanced IL-10 level in UC mice (Figures 6, 7). APE caused the reduction and increase in the inflammatory cytokines of colon and serum in a dose-dependent manner (Figure 6). Furthermore, there was no significant difference in inflammatory cytokine levels between the SASP group and the APE groups (Figure 7).

### APE Treatment Modulated TLR_4_/NF-κB Signaling Pathway in the UC Model

**FIGURE 8:**
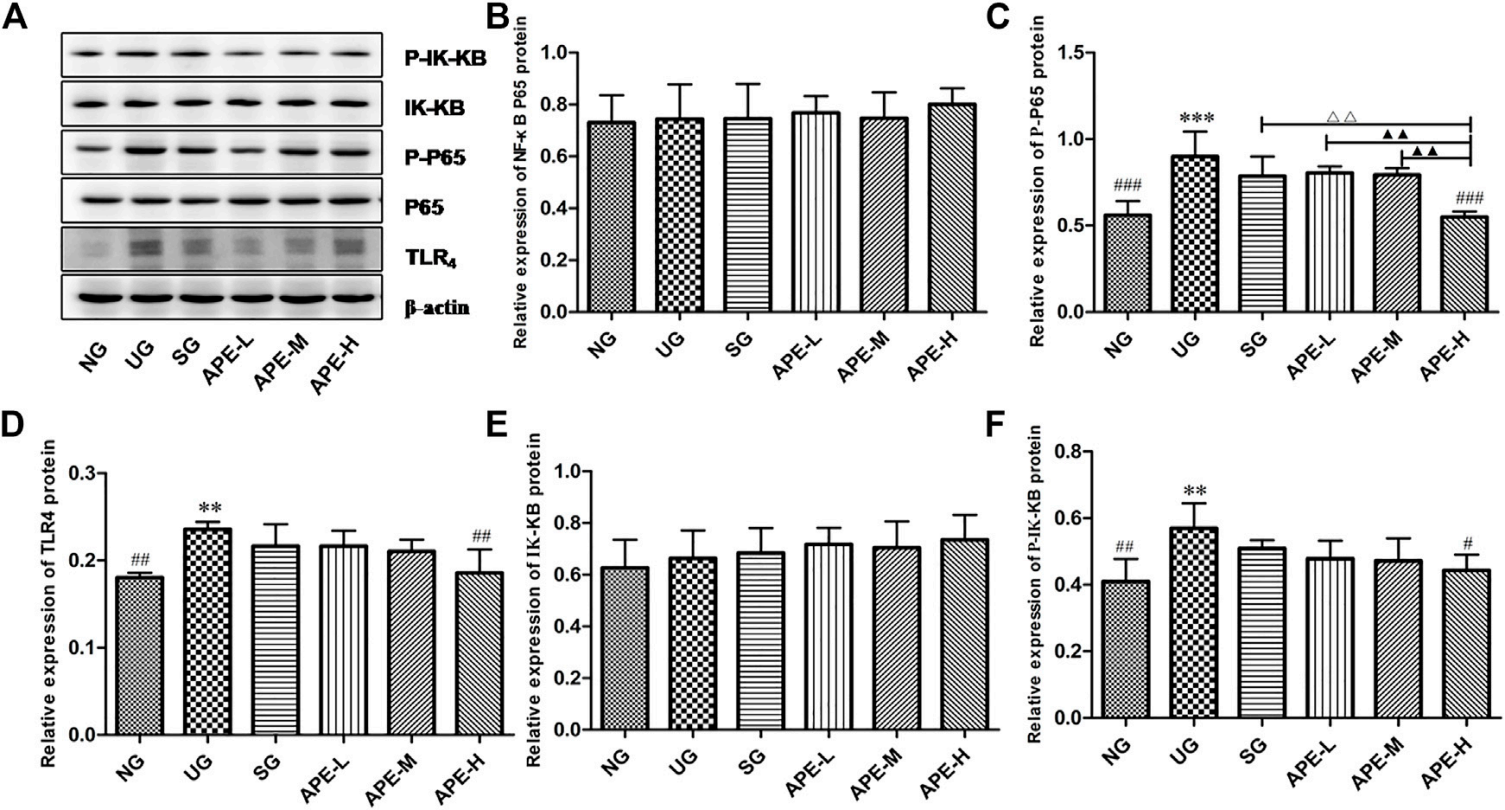
Effects of APE on the levels of relative protein of TLR_4_ dependent NF-κB signaling pathway in the ulcerative colitis mice. **(A)** Western blot analysis demonstrated the effects of APE on the levels of key protein molecules involved in the TLR_4_/NF-κB signaling pathway. **(B)** Relative expression levels of NF-κB. **(C)** p-NF-κB. **(D)** TLR_4_. **(E)** IK-Kβ. **(F)** p-IK-Kβ. *p < 0.05, **p < 0.01, ***p < 0.001 vs. NG. ^#^ *p* < 0.05, ^# #^ *p* < 0.01 vs. UG. ^△^ *p* < 0.05, ^△△^ *p* < 0.01 vs. SG. ^▲^ *p* < 0.05, ^▲▲^ *p* < 0.01 vs. APE-H.NF-κB, nuclear factor-kappa B; TLR_4_,toll-like receptor 4; p-NF-κB, phosphorylation of NF-κB p65; IK-Kβ, inhibitor kappa B-Kinase β; p-IK-Kβ, phosphorylation of p-IK-Kβ; NG, normal group; UG, ulcerative colitis model group. SG,sulfasalazine control group. APE-H,high-dose APE group; APE-M, middle-dose APE group; APE-L, low-dose APE group.

Western blotting was used to investigate the level of TLR4, NF-κB p65, p-NF-κB p65, IK-Kβ, and p-IK-Kβ in UC inflammation to further evaluate the effects of APE. In the present study, the relative protein level of TLR4 was increased in the UG when compared with the NG group (Figure 8D, *p* < 0.01). Similarly, the phosphorylated level of p-NF-κB p65 and p-IK-Kβ were significantly increased in the colon of UC mice compared with that in the NG group (Figure 8C, *p* < 0.001, Figure 8F, *p* < 0.01). More importantly, SASP and APE-H treatment reduced the levels of TLR4 (Figure 8D, *p* < 0.01), p-NF-κB p65 (Figure 8C, *p* < 0.001), and p-IK-Kβ (Figure 8F, *p* < 0.05), and APE treatment caused the changes in a dose-dependent manner. In contrast, the relative protein level of NF-κB p65 was less significant among the UG, SASP, and APE treatment groups. There was a significant difference in the APE-H group compared with the UG group (*p* < 0.05). The changing trend for the ratios of p-NF-κB p65/NF-κB p65 and p-IK-Kβ/IK-Kβ were similar with the changing trend of phosphorylation status of p-NF-κB p65 and p-IK-Kβ. This study suggests that APE treatment modulated the TLR4/NF-κB signaling pathway for fighting intestinal inflammation.

## Discussion

The clinical and pathological manifestations of the DSS model are similar to those observed in human UC (Zhao et al., 2016) and has been used to evaluate the etiology of UC disease and the mechanism of action of drug therapy (Hou et al., 2019). In this study, 2.5% DSS solution was administered to induce UC in ICR mice (Chin et al., 2018). The general characteristics of mice were regularly observed after UC model construction. The results show that the changes in spirit, hair, and diet and observation of diarrhea, even watery stools, and hematochezia were found in the UG group. Additionally, UG group mice displayed symptoms of weight loss, slow growth, increased DAI scores, colon shortening with edema, inflammation, and ulcer (Figures 3A,B). Meanwhile, the CMDI score of colon morphology (Figure 4C), scores of HE and colonic damage (Figure 4D, *p* < 0.001), and the inflammatory responses and factors in the UG group were also increased (Figures 6, 7). These suggested that the establishment of a DSS-induced UC model in mice was successful (Hou et al., 2019). The pathological changes and the high expression of inflammatory factors in the UG group indicated that inflammation was an important factor leading to UC colonic mucosal injury (Liu et al., 2020). However, pathological changes were significantly alleviated after APE and SASP treatment, suggesting that APE may exert a certain effect on the improvement of clinical symptoms and mucosal healing in UC.

In the present experiment, we found that APE treatment gradually relieved the symptoms of diarrhea, blood stool and perianal redness, and swelling. Compared with the UG group, the APE group displayed a significant increase in mice body weight and decrease in DAI scores, colon length reduction (Figure 3), and CMDI scores (Figure 4). APE alleviated the signs of UC damage, including colon inflammation, fossae damage, cell infiltration range of colonic mucosa, pathological changes, and repaired intestinal mucosa injury induced by DSS (Figure 5). The results indicate that APE protects from colon injury and inhibits UC inflammation.

Studies on the pathological mechanism of UC have reported that intestinal inflammation is closely related to multiple targets and pathways in the body, in which TLR_4_ dependent NF-κB is involved in immune regulation and inflammatory expression and plays an important role in the occurrence of disease and intestinal mucosal injury (O'Neill et al., 2009). The TLR_4_-mediated signal transduction pathway is a key link in the pathogenesis of UC inflammation (Gonzalez-Navajas et al., 2010; Zhang et al., 2014). NF-κB is an important multi-gene nuclear transcription factor in the downstream inflammatory signaling pathway of TLR_4_ (Li et al., 2011). NF-κB is the convergence point of multiple signal transduction pathways in the occurrence and development of UC and plays a key role in the genes regulating inflammatory response (Li et al., 2014). To evaluate the inhibitory effects of APE on the TLR_4_ dependent NF-κB signaling pathway, we detected the expression levels of TLR_4_, NF-κB, and Iκβ proteins. In addition, to elucidate the regulatory effect of TLR_4_-dependent NF-κB signals on the inflammatory response of UC, we also detected the levels of inflammatory cytokines IL-1β, IL-6, TNF-α, and IL-10. In this study, as shown in Figure 8, the colonic epithelium of mice in the UG group was highly expressed with TLR_4_ (Figure 8D, *p* < 0.01) when compared with the NG group, and the NF-κB pathway was significantly activated, mainly represented by increased levels of phosphorylated NF-κB p65 and Iκβ protein (Figures 8C,F, *p* < 0.01), which led to the inflammatory response. The levels of inflammatory cytokines IL-1β, IL-6, and TNF-α in peripheral blood serum and colon tissues of UC mice were significantly increased (Figures 6, 7). The results suggest that the TLR_4_-dependent NF-κB signaling pathway is one of the key pathways in the inflammatory response of UC. However, the NF-κB pathway was significantly inhibited in colonic epithelial tissues of UC mice after treatment with SPSA and APE, with significantly reduced levels of phosphorylated NF-κB p65 and IκB protein (Figure 8, *p* < 0.05). Therefore, inhibition of the release of downstream factors in the TLR_4_ signaling pathway may be a molecular mechanism of APE to inhibit the inflammatory expression of UC. Compared with UG group, the levels of pro-inflammatory cytokines IL-1β, IL-6, and TNF-α in serum and colon tissues of UC mice treated with APE were significantly decreased (Figures 6, 7, *p* < 0.05), and the secretion level of anti-inflammatory cytokine IL-10 showed an increasing trend (Figures 6, 7, *p* > 0.05), which indicated that APE may achieve colon mucosal injury by inhibiting the expression of inflammatory cytokines. The inhibition effect of the APE-H group was significantly higher than that of the SPSA group (Figures 6, 7, *p* < 0.05), and there was a dose-effect relationship between the administration group. These showed that APE could inhibit the expression of inflammatory factors downstream of the TLR_4_-dependent NF-κB signaling pathway. Based on the above study results, we speculated that APE may inhibit intestinal inflammation by regulating key proteins and cytokines in the TLR_4_-dependent NF-κB signaling pathway in the intestinal epithelium of mice, thereby reducing intestinal mucosal damage and playing an immunosuppressive role in inflammation.

The discovery of anti-inflammatory active ingredients from natural medicines is a potential way to prevent the progression of UC. As a Uyghur medicine, *Alhagi pseudalhagi* Desv is safe (Ma et al., 2014), economical, and easy to obtain. It has a long history of clinical applications and is mainly used to treat intestinal diseases, relieve abdominal pain, diarrhea, and hematochezia. We speculate that it may play a therapeutic role in the clinical application of UC. We found that the herb extracts contain many natural active ingredients, including flavonoids, polysaccharides, alkaloids, and terpenoids (Quan and Xu, 2009). In the current study, UPLC/MS/MS analysis showed that there were a variety of flavonoid compounds in the main ingredients of APE which were obtained by our previous separation and analysis (Zhang et al., 2010). In addition, we plan to further verify the structure-activity relationship between flavonoid monomers in APE extract and proteins and factors in the NF-κB inflammatory pathway in subsequent experiments. In our study, APE improved the DSS-mediated clinical indicators and pathological changes and inhibited the activation of the TLR_4_-dependent NF-κB inflammatory signaling pathway. Therefore, APE may be a promising drug in the protection of UC and is worthy of further study.

## Data Availability

The datasets presented in this study can be found in online repositories. The names of the repository/repositories and accession number(s) can be found in the article/Supplementary Material.
